# Deepening the Role of Pectin in the Tissue Assembly Process During Tomato Grafting

**DOI:** 10.3390/plants13243519

**Published:** 2024-12-17

**Authors:** Carlos Frey, Susana Saez-Aguayo, Antonio Encina, José Luis Acebes

**Affiliations:** 1 Área de Fisiología Vegetal, Facultad de Ciencias Biológicas y Ambientales, Universidad de León, 24007 León, Spain; cfred@unileon.es (C.F.); a.encina@unileon.es (A.E.); 2 Instituto de Biología Molecular, Genómica y Proteómica, Universidad de León, 24007 León, Spain; 3 Centro de Biotecnología Vegetal, Universidad Andrés-Bello, Santiago 8320000, Chile; susana.saez@unab.cl; 4 Instituto de la Viña y el Vino, Universidad de León, 24009 León, Spain

**Keywords:** degree of methyl-esterification, grafting, homogalacturonan, pectin, pectin methyl-esterase, *Solanum lycopersicum*

## Abstract

Cell walls play essential roles in cell recognition, tissue adhesion, and wound response. In particular, pectins as cell-adhesive agents are expected to play a key role in the early stages of grafting. To test this premise, this study focused on examining the dynamics of the accumulation and degree of methyl-esterification of pectic polysaccharides at the graft junctions using tomato autografts as an experimental model. Monosaccharide analysis showed a marked increase in homogalacturonan from 25% to 32 or 34% at the junction zones early after grafting. In addition, a decrease in the degree of homogalacturonan methyl-esterification up to 38% in the scion and 64% in the rootstock was observed in the first few days after grafting, accompanied by an increase in pectin methyl-esterase activity of up to 20–30% in the tissues surrounding the graft junction. These results shed light on the role of homogalacturonan in grafting and reinforce the key function of pectin as one of the most relevant cell wall components during the grafting process.

## 1. Introduction

Grafting is a horticultural technique in which two living parts of a plant, usually the stem (known as the scion) and the roots (known as the rootstock), are joined to form a single plant with chimera characteristics [1]. The formation of the grafted plant is a dynamic, complex, and continuous process that involves a network of events at the molecular, cellular, tissue, and whole organism levels [2,3]. Many factors contribute to the success of the process, with tissue reunion being a key step [1,4], where the cell wall of the cut edge cells must vary in composition and structure to allow the scion–rootstock attachment [5,6,7]. Plant cell walls are dynamic macromolecules mainly of polysaccharides, with cellulose microfibrils forming a structural scaffold embedded in a functional matrix of hemicelluloses and pectin. Other compounds such as phenolics (i.e., lignin) and structural proteins are also part of the cell wall network [8,9,10].

Among cell wall components, pectins are known to play a crucial role in the reunion of tissues. In fact, pectin domains have been shown to mediate adhesion between plant cells, tissues, and even between plant organs and an inert surface [11,12], including proper graft healing [3]. Pectins consist of a combination of different domains, mainly homogalacturonan (HG) and rhamnogalacturonan I and II [8,9]. These polysaccharides are primarily composed of a backbone enriched in galacturonic acid (GalA), which confers anionic characteristics to them [13]. HG is a linear α-(1–4) GalA homopolymer, where a variable number of GalA residues may be methyl-esterified. Rhamnogalacturonan I is a highly branched and more complex polysaccharide consisting of a backbone of repeating units of GalA and rhamnose disaccharides frequently substituted by lateral chains formed by arabinose, galactose, or arabinose and galactose [13]. HG is the most abundant pectic polysaccharide, constituting 65% of total pectin extracted from the cell wall [14]. Despite its simple linear structure, HG domains play essential roles in cell wall architecture and physiology [15,16]. In fact, HG has been demonstrated to be crucial for maintaining proper cell adhesion in vivo [14]. Recently, an increase in uronic acids (which includes mainly GalA) was reported in tomato graft union tissues close to the cut site several days after grafting (DAG) [17]. In addition, other studies showed the accumulation of HGs in the cut edges of the scion and rootstock of *Arabidopsis* and tomato grafts using the immunolocalization of known epitopes [5,18]. Despite the detection of a putative increase in HG domains at graft union interface as a consequence of grafting, information of the quantitative changes in GalA abundance during this process is lacking in the literature.

Beyond the data obtained through immunolocalization, no additional information has been reported on the properties of HGs during grafting. One of the key characteristics defining HG structure is the degree of methyl-esterification (DME), which represents the proportion of methyl-esters relative to GalA residues [14]. DME is important to define the biological functions of the HG molecule, as a low DME allows the formation of Ca^+2^ bridges (“egg-box” structure) that contribute to the cross-linking of the primary cell wall [13], generating the formation of a micro-gel structure with protective [5] and adhesive features [19] at the scion–rootstock boundary wall, which probably contributes to tissue attachment.

HG is deposited in the cell walls with a high DME and it is subsequently de-methyl-esterified in muro by a set of enzymes called pectin methyl-esterases (PMEs) [20,21,22,23]. During this process, a release of methanol and protons occurs, forming negatively charged carboxyl groups in the process [24]. PME activity is negatively regulated by proteinaceous pectin methyl-esterase inhibitors (PMEIs) [23,25,26]. PME activity, i.e., the balance between PME enzyme yield and PMEI inhibition, is therefore crucial for the determination of HG structure. It can be hypothesized that a high PME activity will lead to extensive HG de-esterification and consequently to the Ca^2+^ bridging of the HG network. Additionally, changes in the pattern of de-esterification have been suggested to influence the fate of HG. Ca^2+^ bridging is more likely to occur upon blockwise de-esterification, whereas random de-esterification is primarily but not exclusively associated with the HG degradation pathway [20,26,27].

In view of the considerations above, the aim of this work was to shed light on the factors surrounding HG accumulation after tomato grafting by studying changes in GalA content, HG methyl-esterification, and PME activity in tissues close to graft union from 0 to 8 days after grafting (DAG). In addition, to determine whether this occurs in non-functional grafts, tissue near the graft junction of these samples was also examined at 8 DAG.

## 2. Materials and Methods

### 2.1. Plant Cultivation

Tomato (*Solanum lycopersicum* L., var. Minibel) seeds (Mascarell Semillas S.L., Valencia, Spain) were sown in 180 mL pots with black peat-based substrate. After seed germination, the seedlings were placed in a growth chamber with a 16/8 light/darkness photoperiod (≈41 µmol m^−2^ s^−1^) and 50–60% of humidity. Plants were watered twice a week with complete Hoagland solution to ≈90% of field capacity [2,17].

### 2.2. Tomato Grafting Method

Approximately one month after germination, when plant stems reached 4–5 mm in diameter, autografting (self-grafted) was performed by cutting at ≈45° the stems below the cotyledonary leaves. To ensure graft union, graft clamps (Toogoo^®^) were placed between the scion and the rootstock. These grafts were maintained at a humidity ≈90–100%, and gradually reduced to ambient humidity in a healing chamber.

### 2.3. Sample Collection and Processing

**Figure 1:**
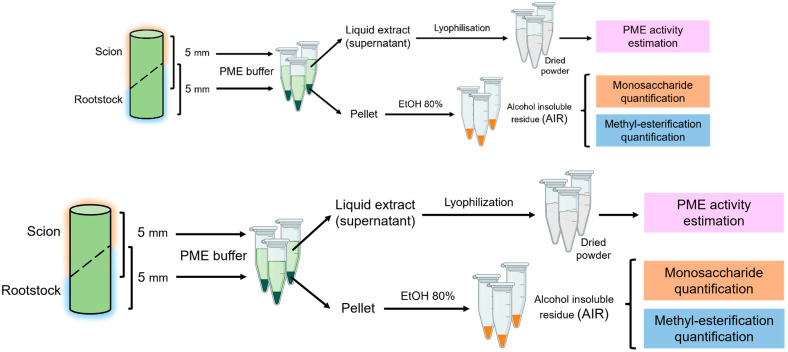
Scheme of sampling and primary treatment of the samples. Note that scion and rootstock were processed separately.

Graft unions (*n* = 5) were collected at different times after grafting: 0 (control), 1, 2, 4, and 8 DAG, including non-functional grafts at this last time. Non-functional grafts were identified by visual inspection of the entire grafted plant. Plants with severe wilting were considered as non-functional after checking the weakness of the graft union. Graft unions were dissected from the stem, also separating the scion from the rootstock, obtaining cylindrical sections of 5 mm in height. These sections were weighed and cold-homogenized in a mortar with pestle using extraction buffer (50 mM Na_2_HPO_4_; 12.5 mM citrate; 1 M NaCl; pH 6.5). After 6 h of gentle shaking in the tubes, the homogenized samples were stored at −80 °C. Then, the samples were thawed and centrifuged at 14,000 rpm for 5 min, and the supernatant was collected and lyophilized. The pellet was washed with 80% ethanol for 5 days and finally dried in a heater at 40 °C for 3 days, obtaining the alcohol insoluble residue (AIR). A summary of the processing method is schematized in Figure 1.

### 2.4. Monosaccharide Quantification

Dried AIR (previously weighed) was incubated with 400 µL 2 N trifluoroacetic acid (TFA) at 121 °C for 1 h with 250 µM myo-inositol and 250 µM allose as internal standards [28]. The samples were then completely dried under air flow at 50 °C, washed twice with 100% 2-propanol, and dried again. Finally, the samples were resuspended in 1 mL of distilled water and filtered using a needle and a filter (0.45 µm of pore size). The filtered samples were analyzed by HPAEC-PAD (high-performance anion-exchange chromatography/pulsed amperometric detection). The hydrolyzed samples were analyzed in a Dionex ICS3000 ion chromatography system equipped with a CarboPac PA1 (4 × 250 mm) analytical column and a CarboPac PA1 (4 × 50 mm) guard column. To separate the different monosaccharides, an isocratic gradient of 20 mM NaOH solution for 32 min, followed by a 75 mM NaOAc and 150 mM NaOH solution for 18 min at a flow rate of 1 mL/min at 26 °C, was used to separate neutral and acidic sugars, respectively. This was followed by a wash with 200 mM NaOH for 5 min. After each run, the column was equilibrated in 20 mM NaOH for 10 min [28]. Standard curves of neutral sugars (d-Fucose, l-Rhamnose, l-Arabinose, d-Galactose, d-Glucose, d-Xylose, and d-Mannose) or acidic sugars (d-Galacturonic acid and d-Glucuronic acid) were used for quantification. Data were expressed as mg monosaccharides/g AIR. The measurements of each sample were carried out with a minimum of four analytical replicates.

### 2.5. Methyl-Esterification Quantification

AIR samples (previously weighed) were resuspended and incubated in 100 µL H_2_O milli-Q and 100 µL 1 N NaOH at 4 °C for 1 h. The reactions were neutralized with 100 µL of 1 N HCl and 300 µL of H_2_O milli-Q was added. Then, the samples were centrifuged at 14,000 rpm for 5 min. Released methanol was measured as described by Saez-Aguayo et al. [25] and Sanhueza et al. [29] with some modifications. (Reaction mix: 50 µL sample supernatant, 100 µL of Tris-HCl buffer, 40 µL of 5 mg/mL methyl-benzothiazolinone-2-hydrazone, and 20 μL of alcohol oxidase. After 20 min of the addition of alcohol oxidase, the samples were incubated with 200 μL of a solution containing 5 mg/mL each of dodecahydrated ferric ammonium sulfate and sulfamic acid, and then diluted with 600 µL of H_2_O milli-Q). The measurements of each sample were carried out with a minimum of five analytical replicates.

### 2.6. Global PME Activity Estimation

Lyophilized samples were resuspended in 1 mL of distilled water and centrifuged at 14,000 rpm for 10 min. Supernatants were used to determine the protein amount with the BCA Kit (Pierce™ BCA Protein Assay Kit, Thermo Scientific, Waltham, MA, USA). Quantification was performed in triplicate for each sample following Saez-Aguayo et al.’s [25] protocol with some modifications. An equal protein amount for each sample in a total volume of 200 µL was loaded into 8 mm diameter wells in the activity gels. The gels consisted of equilibration buffer (50 mM Na_2_HPO_4_ and 12.5 mM citrate, pH 6.5), 0.1% (*w/v*) pectin (from apple, Sigma, Burlington, MA, USA), and 1% (*w/v*) agarose. After 12 h of incubation at 25 °C in the dark, 0.05% (*w/v*) ruthenium red dye was added covering the entire gel surface and slight agitation was applied for 40 min. The gel was then washed three times with distilled water and light pink halos were visible. Once the water was removed, the gels were scanned, and the area was measured using Image J v. 1.53k software.

### 2.7. Statistical Analysis

All data were subjected to Shapiro–Wilk and Levene tests for normal distribution and homoscedasticity checking. Data were subjected to one-way ANOVA using the IBM SPSS Statistics v. 25 software. In addition, Tukey’s test was used for peer comparison. Statistical significance level was set at *p* ≤ 0.05. All results were reported as the mean ± standard deviation (SD).

## 3. Results and Discussion

### 3.1. Decreasing Trend in Monosaccharide Extractability by Acid Hydrolysis

**Figure 2:**
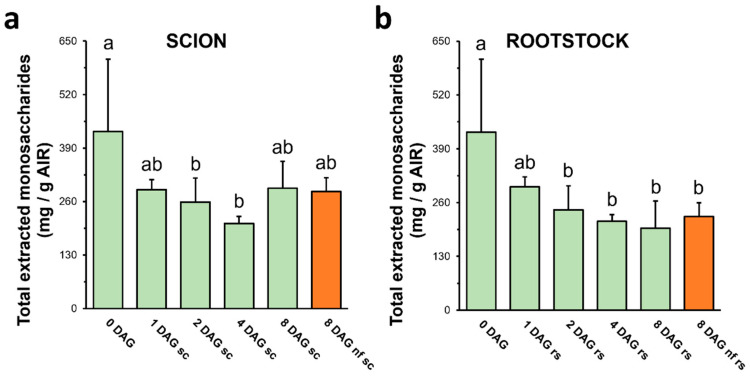
Total extracted monosaccharides of scion (**a**) and rootstock (**b**) after TFA hydrolysis of AIR throughout grafting. Different letters indicate significant differences regard 0 DAG at the *p*-value ≤ 0.05 level after ANOVA followed by Tukey’s test. 0 DAG indicates non-grafted plants at the beginning of the experiment. Bars represent mean ± SD (*n* = 5). The legend “nf” means “non-functional” grafts.

Acid hydrolysis enabled the breakdown of cell wall polysaccharides in the AIR, into their constituent monosaccharides. Monosaccharide recovery depends on the susceptibility of the cell wall to acid hydrolysis and involves a variety of factors such as polysaccharide types (linkage types), polysaccharide cross-linking, and material porosity. The cell wall yield as a result of acid attack varied throughout the grafting process, with lower amounts of monosaccharides being recovered as grafting progressed, even reaching a 50% reduction at 4 DAG in both rootstock and scion (Figure 2). This result could be a consequence of the changes that occur in the cell wall framework as a result of grafting, which would promote the cross-linking of the cell wall polysaccharides, thereby reducing the yield of acid hydrolysis. No difference was observed between functional and non-functional grafts.

### 3.2. Galacturonic Acid Content Increases During Graft Healing Process

**Figure 3:**
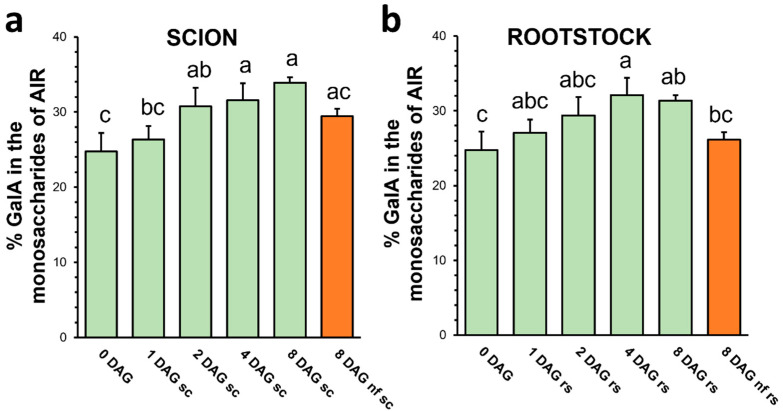
Galacturonic acid (GalA) content of scion (**a**) and rootstock (**b**) throughout grafting after TFA hydrolysis of AIR and quantification by HPAEC-PAD. Different letters indicate significant differences regard 0 DAG at the *p*-value ≤ 0.05 level after ANOVA followed by Tukey’s test. 0 DAG indicates non-grafted plants at the beginning of the experiment. Bars represent mean ± SD (*n* = 5). The legend “nf” means “non-functional” grafts.

HG, the main pectin domain, is a homopolymer formed by a linear chain of partially methyl-esterified GalA. Other pectin domains, such as rhamnogalacturonans, also contain GalA, but it is combined with several neutral sugars [30]. It is therefore expected that an increment in GalA content throughout the grafting process, would indicate an increase in pectins, particularly HG. The results provided here showed that GalA content significantly increased throughout grafting, from 25 to 32 or 34% (Figure 3), indicating an active synthesis and deposition of pectins after grafting (from 0 to 8 DAG) in both scion and rootstock. Consistently with immunolocalization studies [5,18], which show an increase in HG epitopes after grafting, it can be assumed that the increment in GalA mainly corresponds to an increase in HG. It is interesting to note that non-functional 8 DAG plants maintained an intermediate level of GalA between the non-grafted stems and the functional 8 DAG grafted plants. This result suggests that pectin synthesis may occur in non-functional grafts, albeit at a lower intensity, or at least in the early DAG. The de novo synthesis of pectins in non-functional grafts shows that this mechanism, although indispensable for the success of grafting [31], does not ensure its functionality. In the case of tomato, defensive cell wall responses such as lignification and callose or suberin deposition have been demonstrated to drive graft failure [32].

As expected for a process that has been shown to significantly alter matrix polysaccharides [17], other monosaccharides, apart from GalA, also undergo changes during grafting healing (Figure S1). Glucose and xylose decreased during graft healing except in non-functional 8 DAG grafts, and galactose also decreased with time after grafting. On the other hand, arabinose increased from 0 to 4 DAG and rhamnose showed a slight gain from 0 to 8 DAG, suggesting changes in HG and rhamnogalacturonan I structure.

### 3.3. Pectin Methyl-Esterase Activity Showed an Increasing Trend Throughout Grafting

**Figure 4:**
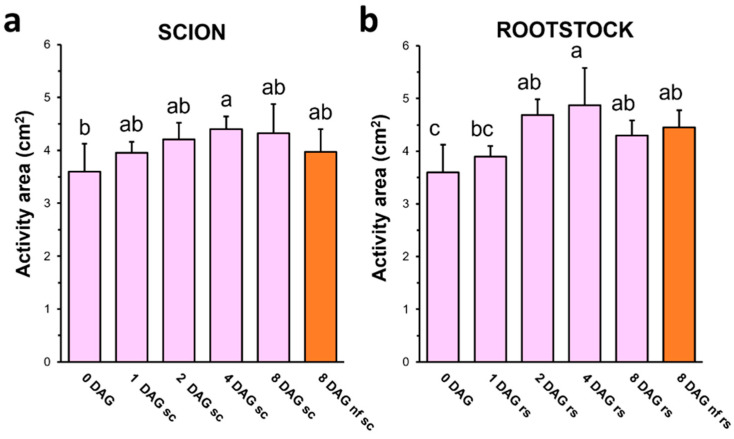
Pectin methyl-esterase enzymatic activity (halo area, cm^2^) of scion (**a**) and rootstock (**b**) extracts throughout grafting. Different letters indicate significant differences regard 0 DAG at the *p*-value ≤ 0.05 level after ANOVA followed by Tukey’s test. 0 DAG indicates non-grafted plants at the beginning of the experiment. Bars represent mean ± SD (*n* = 5). The legend “nf” means “non-functional” grafts.

PMEs are key enzymes in cell wall remodeling. Their activity consists in cleaving the methyl-ester group of GalA, releasing a negative charge that allows Ca^2+^ ions to bind to the polysaccharide and form the “egg-box” structure [30]. Total PME activity at the graft junction was similar in scion and rootstock and increased during grafting up to 20–30% in both tissues (Figure 4). This would suggest a significant role for low-DME HG in the healing process, similar to that found by Sala et al. [5]. On the other hand, PMEs are also involved in the degradation pathway of pectins. In particular, the random (but also blockwise) pattern of de-esterification is associated with HG depolymerization by polygalacturonases and pectin-lyases [20,33]. Pectins are synthesized in their highly methyl-esterified form and the degradation by these latter enzymatic activities only occurs when they are low methyl-esterified [26]. Therefore, the increase in PME activity could not only contribute to the formation of Ca^+2^ bridges, but also to the turnover of the cell wall material, thus promoting the degradation of intensively synthesized pectins.

### 3.4. Total Degree of Methyl-Esterification Showed a Decreasing Trend

**Figure 5:**
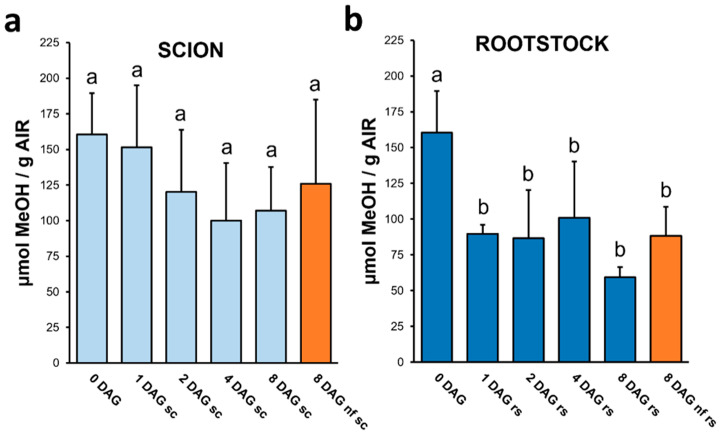
Degree of methyl-esterification of scion (**a**) and rootstock (**b**) throughout grafting. The methanol release was estimated after AIR saponification by a colorimetric method. Different letters indicate significant differences regarding 0 DAG at the *p*-value ≤ 0.05 level after ANOVA followed by Tukey’s test. 0 DAG indicates non-grafted plants at the beginning of the experiment. Bars represent mean ± SD (*n* = 5). The legend “nf” means “non-functional” grafts.

Previous findings indicate that HG with a low DME accumulates in the graft union during healing [5,18]. These studies used antibodies against epitopes of HG with low (such as LM19) and high (such as LM20) methyl groups. DME was also estimated by ATR-FTIR spectroscopy, showing a strong decrease in DME immediately after grafting [17]. However, no study to date has confirmed the changes in DME using a reliable quantitative method of total methyl groups. Here, the methyl-ester groups of the AIR polysaccharides were quantified as methanol-releasing, with DME being higher when more methanol are released [29]. As can be seen, the change trends in DME by grafting are similar in scion and rootstock (Figure 5). In all cases, there was a reduction in DME compared to the control, which was statistically significant in the case of rootstock, reaching a reduction of 38% in scion and 64% in rootstock.

These results show a first quantitative confirmation of the decrease in the DME of pectin at the graft union. Regarding the non-functional grafts, no differences were observed with respect to functional grafts at 8 DAG, demonstrating that the dynamic of the reduction in DME is shared in both non-functional and functional grafts.

**Figure 6:**
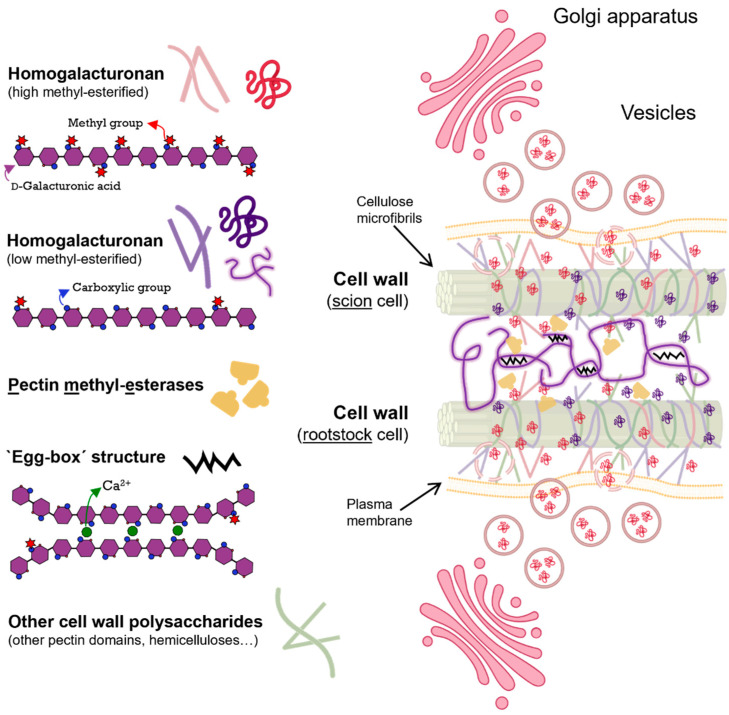
Schematic of the homogalacturonan-mediated cell wall adhesion hypothesis in plant grafts. Esterified homogalacturonan is transported de novo from the Golgi apparatus to the cell wall, where it is de-esterified by pectin methyl-esterase activity, which is expected to allow the formation of the ‘egg-box’ structure, which is assumed to confer special adhesive properties on the new graft cell wall interface. Made with BioRender and Inkscape v. 1.4.

In this work, we report solid data on the accumulation of GalA in the graft union during the graft healing process, accompanied by an increase in PME activity (a balance between PME enzyme activity and its inhibitors) and a decrease in DME, estimated by methanol release. These results, together with the background in the literature [5,17,18], could lead us to propose the hypothesis of HG-mediated cell wall adhesion in plant grafts, schematized in Figure 6.

Given that graft healing implies an increase in HG and a decrease in DME, it is hypothesized that the inversely correlated decrease in acid hydrolysis yield observed (see Figure 2) could be a consequence of the pectin increment. It is known that at low DME, HG has the ability to bind Ca^2+^ and create gel-like structures (“egg-boxes”), which, in turn, influence the rheological properties of the cell wall [34,35], as they have adhesive features [19], contribute to primary cell wall cross-linking [13], and increase cell wall stiffness [36,37]. The increment in the cross-linking and the stiffness could explain the resistance to the acid attack (and therefore the decrease in hydrolysis yield). Another hypothesis is that the reduction in acid hydrolysis yield is only due to a relative effect of increased HG in the cell wall. The GalA bond is partially resistant to acid hydrolysis, implying that the release of GalA from HG requires more demanding hydrolysis conditions, and, on the other hand, GalA can be degraded to lactones after hydrolysis [38]. This could explain the decrease in cell wall acid hydrolysis as only an increase in this pectin. Another reason for this result could be the accumulation of polymers resistant to acid hydrolysis, such as cellulose or lignin.

To summarize, this study has deepened the quantitative and qualitative changes in HG throughout the grafting process, observing (1) an increase in GalA; (2) a rise in PME activity; and (3) a decreasing trend of DME in scions and a clear decrease immediately after grafting in rootstocks. However, further studies are needed as the changes that occur during healing make this system very complex to study and some properties and physiological effects of de-esterified HG are not yet fully understood. Indeed, studies on finer changes in the structure of HG and also of rhamnogalacturonan cannot be excluded and need to be evaluated.

## Data Availability

The data in the original article are available on request from the corresponding author, due to the nature of the investigation.

## Supplementary Materials

The following supporting information can be downloaded at: https://www.mdpi.com/article/10.3390/plants13243519/s1, Figure S1: Monosaccharide profile (including fucose, rhamnose, arabinose, galactose, glucose, mannose, xylose, galacturonic acid and glucuronic acid) of scion (sc) and rootstock (rs) tissues of the graft union from 0 to 8 days after grafting (DAG), including 8 DAG non-functional (nf) grafts.
