# Supplementary material for: Deepening the Role of Pectin in the Tissue Assembly Process During Tomato Grafting
Source: Plants (Basel). 2024 Dec 17;13(24):3519. doi: 10.3390/plants13243519 (PMC11677993; doi:10.3390/plants13243519)
Supplement: Supplementary file 1 [file plants-13-03519-s001.zip › plants-3341972-supplementary.pdf]

Supplementary material

Deepening the role of pectin in the tissue assembly process during grafting

Carlos Frey[1,2], Susana Saez-Aguayo[3], Antonio Encina[1,2] and José Luis Acebes[1,4*]

[1]Área de Fisiología Vegetal, Facultad de Ciencias Biológicas y Ambientales, Universidad de León, León, España.

[2]Instituto de Biología Molecular, Genómica y Proteómica, Universidad de León, León, España.

[3]Centro de Biotecnología Vegetal, Universidad Andrés-Bello, Santiago, Chile.

[4]Instituto de la Viña y el Vino, Universidad de León, León, España.

*Correspondence: JLA (jl.acebes@unileon.es; 0000-0002-0960-085X). Campus de Vegazana, 24007, León (Spain).

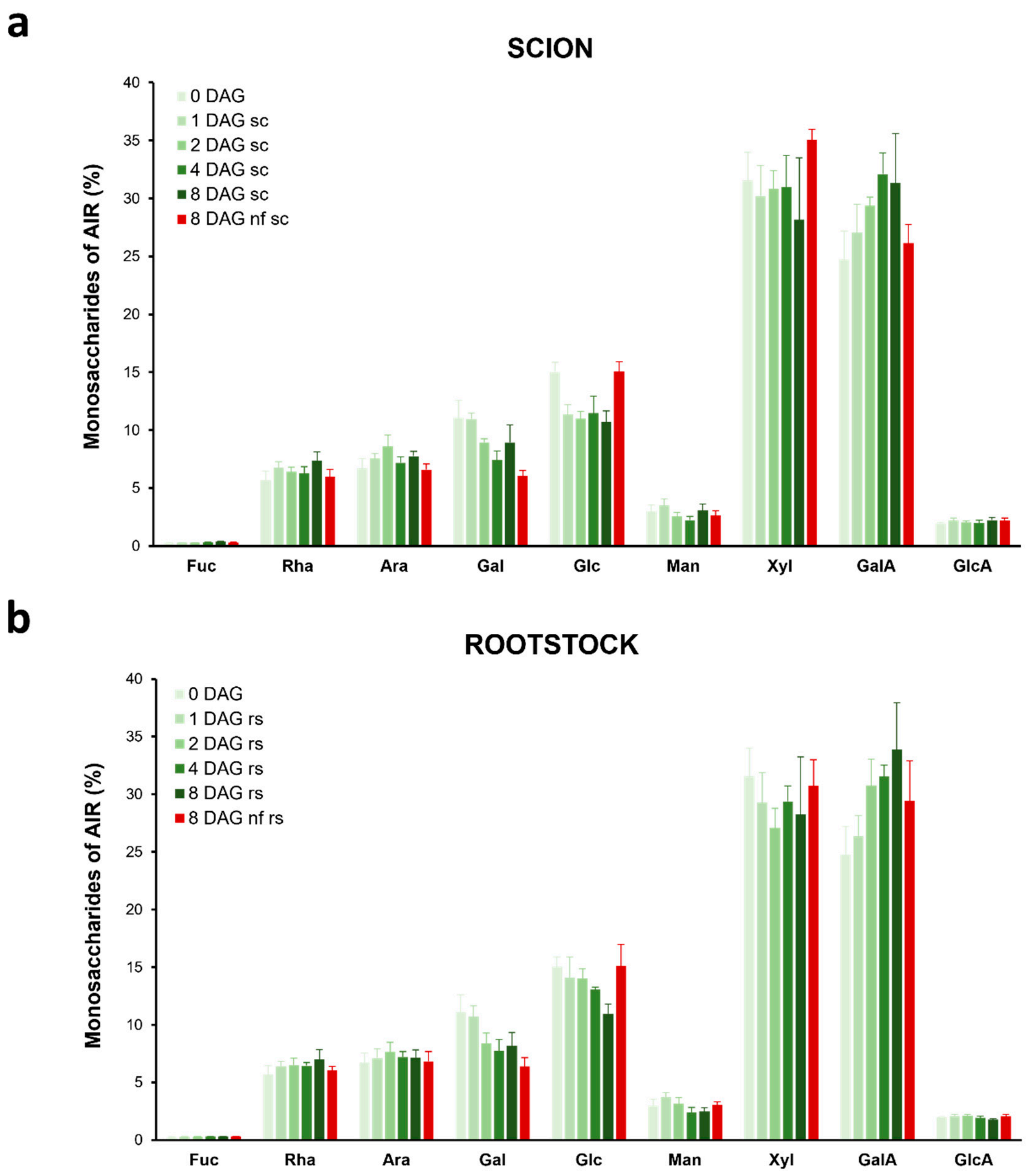

Figure S1 Monosaccharide profile (including fucose, rhamnose, arabinose, galactose, glucose, mannose, xylose, galacturonic acid and glucuronic acid) of scion (sc) and rootstock (rs) tissues of the graft union from 0 to 8 days after grafting (DAG), including 8 DAG non-functional (nf) grafts.
